# Cellular and Molecular Responses to Mechanical Expansion of Tissue

**DOI:** 10.3389/fphys.2016.00540

**Published:** 2016-11-15

**Authors:** Muhammad Abdur Razzak, Md. Sanower Hossain, Zamri Bin Radzi, Noor Azlin B. Yahya, Jan Czernuszka, Mohammad T. Rahman

**Affiliations:** ^1^Department of Children's Dentistry and Orthodontics, Faculty of Dentistry, University of MalayaKuala Lumpur, Malaysia; ^2^Department of Materials, University of OxfordOxford, UK

**Keywords:** tissue expansion, growth factors, focal adhesion complex, apoptosis, ion channels, secondary messengers

## Abstract

The increased use of tissue expander in the past decades and its potential market values in near future give enough reasons to sum up the consequences of tissue expansion. Furthermore, the patients have the right to know underlying mechanisms of adaptation of inserted biomimetic, its bioinspired materials and probable complications. The mechanical strains during tissue expansion are related to several biological phenomena. Tissue remodeling during the expansion is highly regulated and depends on the signal transduction. Any alteration may lead to tumor formation, necrosis and/or apoptosis. In this review, stretch induced cell proliferation, apoptosis, the roles of growth factors, stretch induced ion channels, and roles of second messengers are organized. It is expected that readers from any background can understand and make a decision about tissue expansion.

## Introduction

Since the first utilization in 1957 (Neumann, 1957), the use of tissue expansions have become widespread in maxillary and craniofacial surgery (Kobus, 2007), burn scar excision (Hafezi et al., 2009), breast reconstruction following mastectomy (Lohsiriwat et al., 2013), ophthalmology (Hou et al., 2012), management of omphalocele (Clifton et al., 2011), nasal reconstruction (Kheradmand et al., 2011), scalp alopecia (Guzey et al., 2015) and other deformities in plastic reconstructive surgery (Motamed et al., 2008; Laurence et al., 2012; Santiago et al., 2012). Tissue expander generates new tissues, by exploiting the viscoelastic properties of the skin and adjusted histological changes which follows the principle of the controlled mechanical skin overstretch (Argenta, 1984; Pamplona et al., 2014). It involves the insertion of a biomimetic and bioinspired material (i.e., hydrogel tissue expander) adjacent to a wound or defect that needs to be resurfaced (Motamed et al., 2008; Swan et al., 2012). The expanded tissue can then be used to resurface a defect or incorporate permanent prostheses (Kasper et al., 2012; Swan et al., 2012).

Nevertheless, tissue expansion for the reconstructive surgery are also associated with a variety of complications (Adler et al., 2009; Huang et al., 2011). Swan et al. (2012) observed mucoperiosteal ulceration while using uncoated self-inflating anisotropic hydrogel tissue expander in the porcine hard palate. Minor side effects on skin histology and circulation resulted in skin stretching with staples or hypodermic needles, thus proving the Pavletic device to be non-feasible in primary wound closure (Tsioli et al., 2015). Incidence of infection, being the most common complication (Huang et al., 2011), has witnessed a total of 16 cases out of 215 children who underwent reconstruction with tissue expanders (Adler et al., 2009). However, the pivotal concern is to ensure normal tissue patterning and prevent tumor or scar formation (Huang and Ingber, 1999; Aarabi et al., 2007).

**Figure 1:**
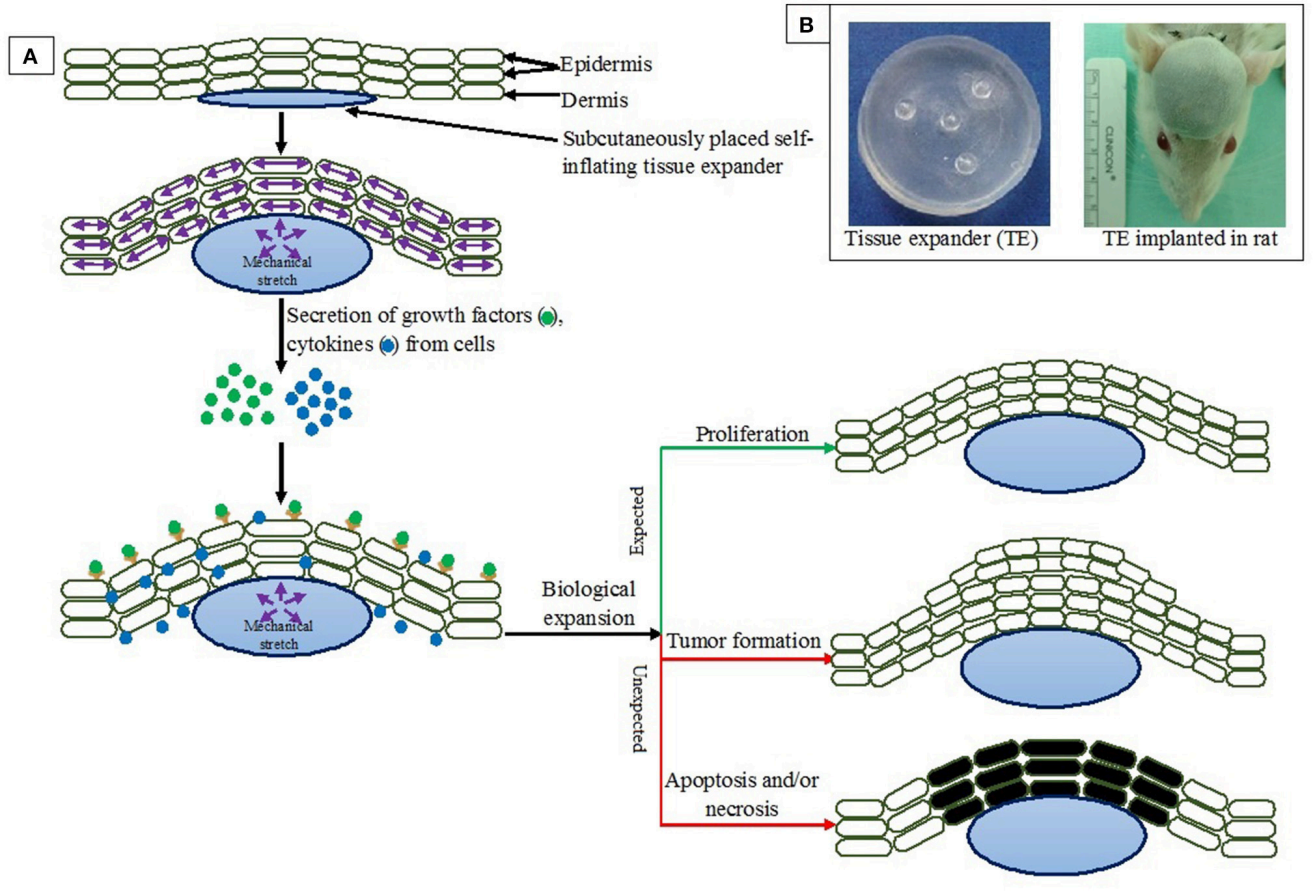
**(A)** Effects of tissue expansion on surrounding tissues. **(B)** Tissue expander before implantation and implanted in rat. Pictures taken from ongoing research in author's lab.

Recent studies revealed that rapid changes in extension, alignment, and collagen adapt to mechanical expansion (i.e., stretch or strain). Both elastin and collagen realign in a parallel fashion in response to stretch and/or expansion (Verhaegen et al., 2012; Tsioli et al., 2015), and the elongation occurs to the direction of stretching (Figure 1). Mechanical stretch on tissue is related to several physiological phenomena such as cellular growth enhancement and/or expansion with a significantly higher vascularity of expanded tissue (Yano et al., 2004). Strain beyond physiological limit may lead to alteration of cell function such as tumor formation, necrosis and/or apoptosis (Chen et al., 1997; Huang and Ingber, 1999; Wernig et al., 2003; Knies et al., 2006). Hence lies the clinical implications of tissue expansion (Swenson, 2014; Kwon et al., 2016).

In physiological condition, tissue development and remodeling are highly regulated. A number of studies have focused on the cellular and molecular mechanisms (such as integrated network of cascades, implicating growth factors, cytoskeleton, protein kinase family, synthesis of DNA, expression of gene) leading to the increase of skin surface area (Plenz et al., 1998; Takei et al., 1998; Skutek et al., 2003; Knies et al., 2006; Jaalouk and Lammerding, 2009; Wong et al., 2011; Wu et al., 2015). Under mechanical stress, the cell phenotype and the nature of the physical stimuli determine which signal transduction pathways are activated during tissue expansion (Hsieh and Nguyen, 2005). This review, will focus the reports of molecular events of skin-derived cells in response to mechanical strain. The response of cells to mechanical stretch, the roles of growth factors, effects on extracellular matrix, cell membrane, and stretch induced ion channels, roles of second messengers, and cellular interactions will be organized from the extracellular to intracellular pathways with future perspectives in the conclusion.

## Response of cells to mechanical stretch

The viscoelastic properties of skin to increase surface area in response to forces are the basic biology of tissue expansion (Bascom and Wax, 2002). The external forces are transmitted through the multi-layered skin which consists of epidermis connected to the dermis and the underlying subcutaneous tissues (Schwartz and DeSimone, 2008). The morphological and physiological consequences of tissue expansion on various layers of skin and other cellular and muscular components are summarized in Table 1.

**Table 1:** **Responses of tissues to expansion**.

**Tissues**	**Effects observed**	**References**
Epidermis	Increased density and thickness of epidermis up to 40% instead of normal state (10%) in expanded skinReduced intercellular spaces in all layers of the epidermis Remarkably increased the mitotic activity of epidermis; resulting increased DNA synthesis and therefore cellular proliferation Maintained phenotypical characteristic of epidermis	Austad et al., 1982; Vander Kolk et al., 1987; van Rappard et al., 1988; Silver et al., 1992.
Dermis	Thinned dermal thickness rapidly with an average of 20% and thickness may return to normal within 2 years following expansion Decreased the density of hair follicles in the expanded skin but quantitatively and functionally remain unchanged Increased collagen synthesis in the dermis during tissue expansion Observed temporary hyperpigmentation in expanded tissue upon up-regulation of melanin expression during tissue expansion	Austad et al., 1982; Pasyk et al., 1988; Johnson et al., 1993.
Fat	Lost subcutaneous fat permanently Decreased the thickness of adipose tissue and markedly decreased the number of fat cells by as much as 30 to 50% May flattened or disappeared adipocytes altogether during the expansion process Occurred a varying amount of fat necrosis during tissue expansion process, the degree of which is related to the rate of expansion	Leighton et al., 1988; Pasyk et al., 1988; Takei et al., 1998.
Muscle	Sensitive to tissue expansion and changed ultra-structural Thinned muscle in expanded skin without changing the number of cells Increased number and size of mitochondria, number of vesicles and amount of sarcoplasm Undergo atrophy and weakness after expansion resulting in the so-called bath-tub depression, but permanent sequelae are rare	Pasyk et al., 1982; Sasaki and Pang, 1984; Stark et al., 1987; Johnson et al., 1993.
Capsule	Developed a dense fibrous capsule around the expander after few days of implantation Elongated fibroblasts, which stimulates the synthesis of collagen Developed double-layered capsule within 7 days of expander implantation Increased the thickness of capsule after 2 to 2.5 months of expansion	Austad et al., 1982; Johnson et al., 1993.
Blood vessels	Observed rapid angiogenesis and distention of capillaries during expansion Increased the number of arterioles and venules within few days of expansion Elongated veins and arteries rapidly with no loss of diameter or intimal integrity	Sasaki and Pang, 1984; Stark et al., 1987; Saxby, 1988.
Nerve	Nerve tissue is tolerant to tissue expansion and no demyelination or necrosis of nerve tissue Lengthen the peripheral nerve without significant damage No neurologic change in response to expansion during tissue expansion (Intraluminal pressure more than 44 mm Hg may cause reduction of axon potential)	Swenson, 2014.
Bone	Tissue expansion causes significant but reversible cranial and long bone changes Reduced bone thickness and volume during tissue expansion Noticed erosion beneath the expander without changing bone density Nothing changed in the inner table of the skull or stigmata	Antonyshyn et al., 1988; Moelleken et al., 1990; Johnson et al., 1993.
Vascular plexus	Enhanced angiogenesis in expanded tissues might be caused of increased gene expression and VEGF level Raised more vascularized flaps in expanded tissue and survived to a greater length, averaging 117% over control flaps	Saxby, 1988; Nikkhah et al., 2015.

**Figure 2:**
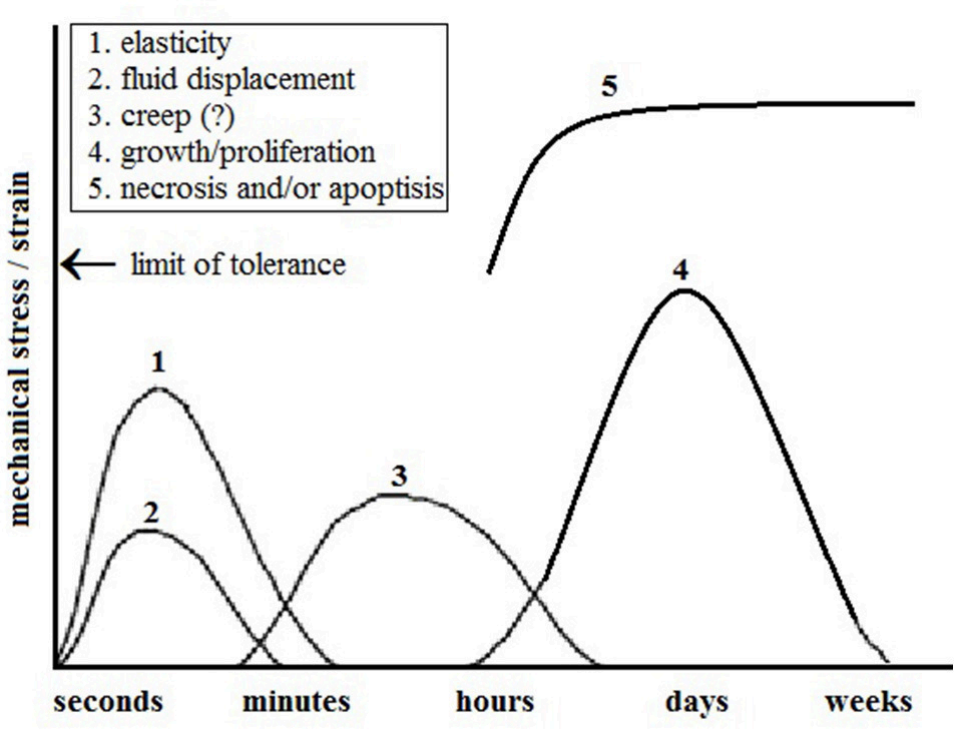
**Physiological and cellular response of skin to mechanical stress**. It is most likely that mechanical stress beyond the limit of tolerance of elasticity might induce necrosis and/or apoptosis at the cellular level (Modified from Siegert et al., 1993).

Numerous researchers have linked the mechanisms that lead to an increased length with skin's elasticity (Kenedi et al., 1975; Bader and Bowker, 1983; Larrabee Jr and Sutton, 1986). Gibson et al. (1965) associated the increase in skin length with the interstitial displacement of fluids and skin's creep behavior. Austad et al. (1982) reported that the increased length was as a result of cellular proliferation. Siegert et al. (1993) simplified these findings relating the strain, time and mechanism of skin expansion as shown in Figure 2. Because of its elasticity, the skin expands practically without temporal delay after expansion pressure is exerted. Interstitial displacement of fluids can be seen (in oedema) after skin expansion. Larrabee Jr et al. (1986), Gibson et al. (1965) and Wilhelmi et al. (1998) suggested that the biological creep (i.e., the generation of new tissue) is due to the chronic stretching forces. It is also most likely that similar events such as interstitial fluid displacement and elasticity beyond the tolerance limit of the tissue might induce necrosis and/or apoptosis of the tissue (Linder-Ganz and Gefen, 2004).

**Figure 3:**
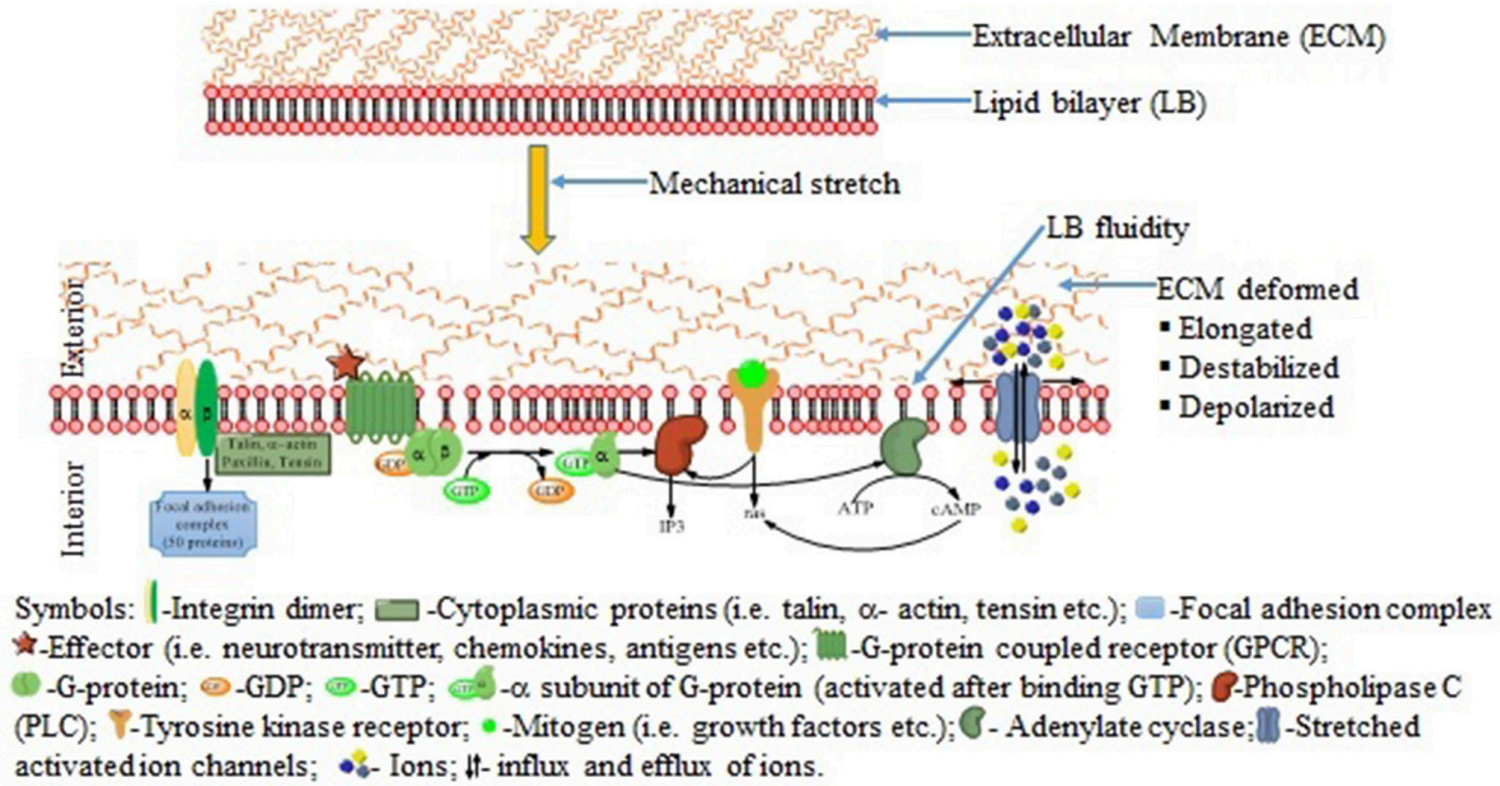
**Possible signaling pathways activated in response to mechanical stretch**. Upon application of forces, extracellular matrix deformed and the plasma membrane altered resulting activation of the ion channel and activate the integrins, G-protein coupled receptors, tyrosine kinase receptors and others membrane bound signaling pathways.

Cell stretching, in some contexts causes apoptosis, and in others promotes cell proliferation (Takei et al., 1998; Skutek et al., 2003). Similarly, apoptosis and proliferation pathways share many common elements, and they converge and influence each other at different levels (Wernig et al., 2003). Application of mechanical stretch (stimulus) activates mechanosensitive ion channels, G-protein coupled receptors, protein kinases, integrin-matrix interactions and other membrane-associated signal-transduction molecules to convert physical cues to biologic responses (Schwartz and DeSimone, 2008; Jaalouk and Lammerding, 2009) (Figure 3).

### Stretch induced proliferation

**Figure 4:**
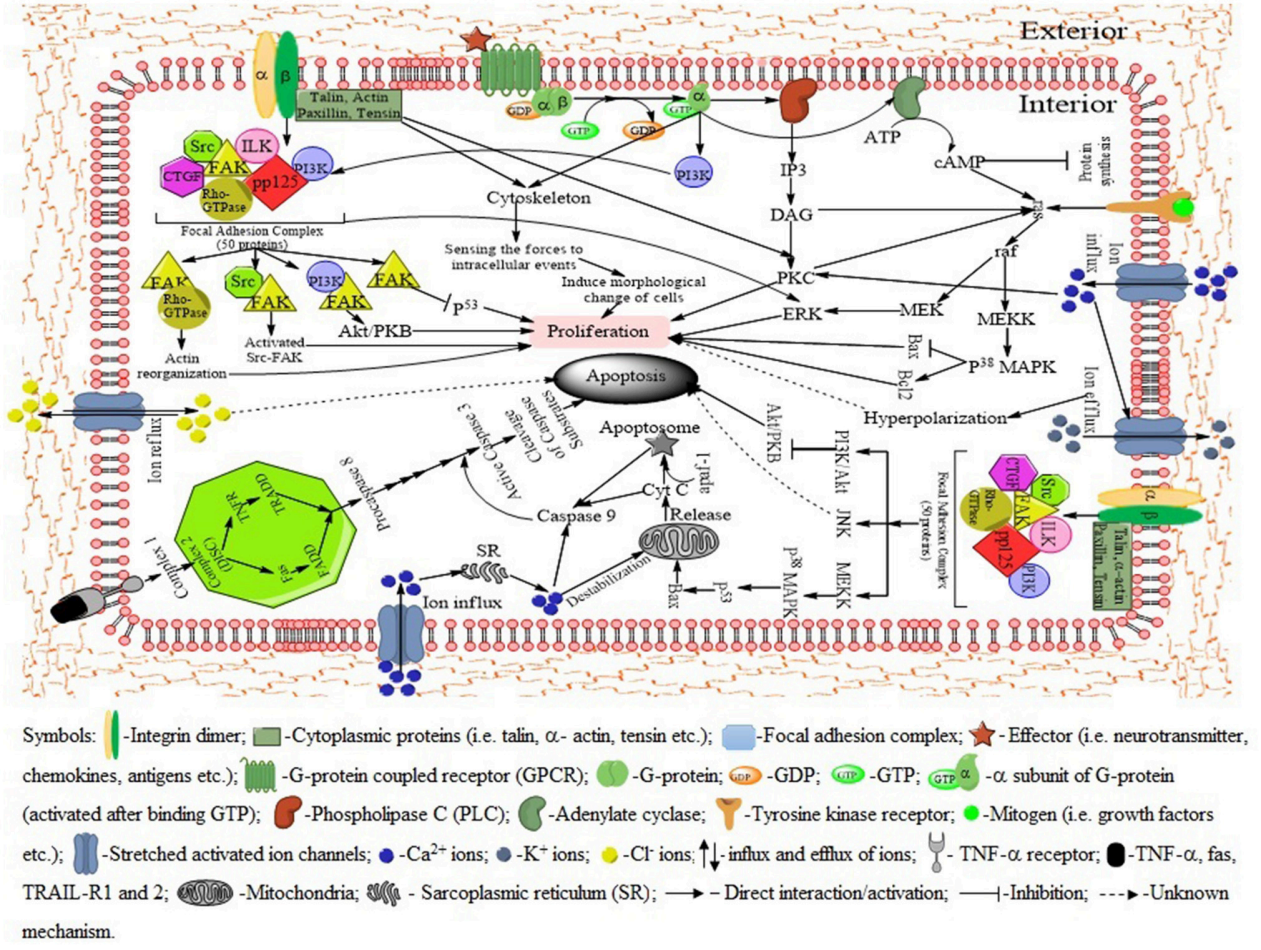
**Signaling pathways activated by mechanical stretch leading to either cell proliferation or apoptosis**. The integrins organize the cytoskeleton according the physical properties of the extracellular matrix (ECM). The membrane bound ion channels, G-protein, tyrosine kinase receptor and other molecules activate specific pathways to proliferation. In case of apoptosis, receptor-like molecules such as integrins, focal adhesion proteins become activated and these molecules in turn activate a limited number of protein kinase pathways (p38 MAPK, PI3K/Akt, JNK etc.), which amplify the signal and activate enzymes (caspases) that promote apoptosis. Activation of death receptors (Fas and/or TNFR) leads to the formation of a death-inducing signaling complex (DISC), resulting in the cleavage of procaspase-8 to its active form. Caspase-8 in turn activates downstream proteins that lead to apoptosis. Bax, induces the release of cytochrome c from the mitochondria and promotes apoptosis. Moreover, cytochrome c complexes with apaf-1 and procaspase-9 to form an apoptosome. This leads to the activation of caspase-9, which in turn activates effector caspases (3, 6, and 7) and subsequent apoptosis. Among the stretch-activated ion channels, rapid influx of Ca^2+^ activate several pathways including signal transduction cascades leading to cell proliferation, apoptosis, cell contraction, activation of potassium channel. Potassium channels play roles in maintaining optimal membrane potentials. Mechanical forces and calcium influx also open chloride channels which act as apoptotic agents through a delineated mechanism.

In response to mechanical stretch, cells of the cutaneous tissues, such as fibroblasts, receive the signals and prepare to proliferate (Silver et al., 2003). The extracellular matrix (ECM) plays a central role in strain-induced cell proliferation (Hynes, 2002). The extracellular forces transmitted through the ECM leading to the deformation of the matrix, followed by alteration of plasma membrane and adhesion complexes (Chien, 2007). The transmembrane protein integrin communicate with both extracellular matrix and cytoplasmic proteins such as talin, paxilin, and vinculin. Integrins also sense the physical properties of the ECM and organize the cytoskeleton accordingly (Zamir and Geiger, 2001). Binding of talin to the integrin cytoplasmic tail induce a conformational change from an inactivated to an activated state with an increase affinity for the ECM (Tadokoro et al., 2003). Upon the activation of integrins, the β subunit complexes with numerous structural and signaling proteins to form a focal adhesion complex (FAC) to provide both the physical link between integrin-adhesion receptors and the actin cytoskeleton, as well as sites of signal transduction into the cell interior (Carragher and Frame, 2004; Wozniak et al., 2004). The activated FAC then activate signal transduction pathways that co-ordinate cell proliferation (Figure 4). Hence it is well evident that a number of growth factors in ECM regulate cell proliferation (Singh et al., 2009; Bush and Pins, 2010).

Recently, Jiang et al. (2016) demonstrated that, static stretch conditions can increase collagen I levels but decrease fibronectin levels compared to a cyclic stretch conditions where collagen I is significantly reduced but fibronectin is markedly increased. Thus, cyclic stretch suppressed human fibroblast proliferation compared to that with static stretch. Again, nuclear envelope proteins such as emerin or lamin A/C were shown to play critical roles in suppressing vascular smooth muscle cells hyperproliferation induced by hyperstretch (Qi et al., 2016).

### Stretch induced apoptosis

A balanced cell proliferation/growth and apoptosis is a pre-requisite for normal development and for adaptation to a changing environment (Jacobson et al., 1997). Too little apoptosis can promote cancer and autoimmune diseases; whereas, too much apoptosis can augment ischaemic conditions and drive neurodegeneration (Czabotar et al., 2014). Apoptosis can be triggered either by external receptor-dependent stimuli (ligation of death receptors with their cognate ligands, such as FasL, TRAIL or TNF) or internal mitochondria-mediated signaling (Adams, 2003; Özören and El-Deiry, 2003).

Different stimuli such as intracellular damage, cytotoxic compounds and developmental activates the mitochondrial (intrinsic) pathway of apoptosis (Liao et al., 2004, 2005). In this pathway, stretch activates pro-apoptotic effectors Bax and Bak, which then disrupt the mitochondrial outer membrane resulting in the release of cytochrome c (Figure 3). Cytochrome c then leads to the formation of the apoptosome with the help of apoptotic protease-activating factor 1 (apaf-1) that promotes caspase 9 activation (Li et al., 1997; Luo et al., 1998; Zou et al., 1999). In the death receptor-mediated pathways (extrinsic) of apoptosis, certain death receptor ligands of the tumor necrosis factor (TNF) family (such as Fas ligand and TNF) bind with their cognate death receptors (FAS and TNFR1, respectively) on the plasma membrane, leading to caspase 8 activation via the Fas-associated death domain protein (FADD) and the TNFR-associated death domain protein (TRADD) in a cytosolic death-inducing signaling complex (DISC) also known as complex II (Wang et al., 2008; He et al., 2009). These two pathways converge at activation of the effector caspases (caspase 3, caspase 7, and caspase 6) (Adams, 2003).

Necrosis, known as a catastrophic form of death, is typically not associated with caspases activation and mediates cells' demise in response to severe injuries or in case of a pathological evet (Vanden Berghe et al., 2004). Although, apoptosis and necrosis may occur simultaneously in response to specific stimuli, the morphological characteristics of cell undergoing necrosis are distinct from those seen in cells undergoing apoptosis (Kroemer and Levine, 2008). However, mechanisms of necrosis due to tissue expansion are not fully understood.

## Major roles of growth factors in tissue expansion

The cellular growth, tissue integrity and eventually the reestablishment of the barrier function of the skin is executed and regulated by the coordinated efforts of several cell types (keratinocytes, fibroblasts, macrophages, platelets etc.) and numerous growth factors (biologically active polypeptides) (Werner et al., 2007; Gurtner et al., 2008). The epidermal growth factor (EGF) family, transforming growth factor beta (TGF-β) family, fibroblast growth factor (FGF) family, vascular endothelial growth factor (VEGF), platelet derived growth factor (PDGF), connective tissue growth factor (CTGF), interleukin (IL) family are all important in stress (either mechanical or physiological) induced cell growth (Werner et al., 1994; Shimo et al., 1999; Steiling and Werner, 2003; Shirakata et al., 2005; Secker et al., 2008). The functions of growth factors depend on source and binding with specific receptors and can act by paracrine, autocrine, juxtacrine, and endocrine mechanisms (Barrientos et al., 2008). Earlier studies showed that, EGF, FGF-2, TGF-β, PDGF, and VEGF levels are increased in early after injury and decreased at chronic states and IL-1 and 6, and TNF-α levels increased both in early and chronic states (Brown et al., 1986; Frank et al., 1995). The functions of various growth factors are summarized in Table 2.

**Table 2:** **Growth factors in response to mechanical or physical stress on different tissues**.

**Growth factor**	**Native cells**	**Experimental condition (expansion or stress)**	**Effect on growth factor**	**Major observations**	**References**
Epidermal growth factor (EGF)	Macrophages, Fibroblasts	Burn injuries	↑	Keratinocyte proliferation and migration	Grayson et al., 1993
		2 mm incisional wounds on the PU.1 null mouse	↑	Reepithelialisation	Martin et al., 2003
Heparin-binding epidermal growth factor (HB-EGF)	Macrophages	Keratinocyte-specific HB-EGF-deficient mice	↓	Wound closure was markedly impaired	Shirakata et al., 2005
		Cells treated with tetracycline (TET)	↑↑	Overexpression of HB-EGF inhibits proliferation	Stoll et al., 2012
Fibroblast growth factor 1, 2, and 4 (FGF 1, 2, and 4)	Fibroblasts, Macrophages, Endothelial cells, Smooth muscle cells, Chondrocytes, Mast cells	Cultured fibroblasts stimulated with IL-1α	↑	Fibroblast proliferation Angiogenesis	Maas-Szabowski and Fusenig, 1996
Transforming growth factor-α (TGF-α)	Macrophages, Keratinocytes	Macrophages isolated from a wound site	↑	Keratinocyte migration and reepithelialisation	Rappolee et al., 1988
Transforming growth factor-β1-3 (TGF-β1-3)	Macrophages, Fibroblasts, Keratinocytes, Neutrophils	Adult and fetal wounds	II ↑	Reepithelialisation of skin Epidermal differentiation	Cowin et al., 2001a
		Fetal and adult sheep incisional skin wounding	↑	TGF-β3 is anti-scarring	Scheid et al., 2002
Amphiregulin (AR)	Keratinocytes	Serum free cultured human keratinocytes	↑	Keratinocyte proliferation	Piepkorn et al., 1994
Keratinocyte growth factor (KGF or FGF7)	Fibroblasts	Wounded mice skin	↓	Delayed re-epithelialization due to reduced proliferation rate of epidermal keratinocytes	Werner et al., 1994
Platelet derived growth factor (PDGF)	Macrophages, Endothelial cells	Acute incisional wounds in an aging mouse colony	↓	The low levels of PDGF in the old cause initial delay in fibroblasts and inflammatory cell infiltration and proliferation within the wounds	Ashcroft et al., 1997
Hepatocyte growth factor (HGF)	Mesenchymal cells, Hepatocytes, Adipocytes, Keratinocytes	Adult rat excisional wounds	↑	Keratinocyte migration, and proliferation Angiogenesis	Cowin et al., 2001b
Vascular endothelial growth factor (VEGF)	Neutrophils, Macrophages, Endothelial cells, Fibroblasts,	Immobilized VEGF in porous collagen scaffold	↑	Endothelial cell proliferation, migration, and angiogenesis	Shen et al., 2008
Connective tissue growth factor (CTGF)	Fibroblasts, Endothelia	Scratched human corneal epithelial cells	↑	CTGF is strongly induced and caused pathophysiology in tissues by inducing matrix deposition, conversion of fibroblasts into contractile myofibroblasts	Secker et al., 2008
Insulin-like growth factor-I (IGF-I)	Fibroblasts, neutrophils, macrophages, hepatocytes and skeletal muscle	Estrogen-deprived mice	↑	Keratinocyte and fibroblast proliferation and migration Collagen synthesis and re-epithelialization	Emmerson et al., 2012
		Rat surgical incision	↑	Re-epithelization	Todorovic et al., 2008
Interleukin-I α and β (IL-I α and β)	Neutrophils, Monocytes, Macrophages, Keratinocytes	Irradiated fibroblasts	↑	Keratinocyte activation, migration and proliferation Induce KGF expression and fibroblasts creation	Maas-Szabowski et al., 2000
Endothelin-I (ET-I)	Keratinocytes, Fibroblasts, Endothelial cells	Cyclic stretch of cultured rat aortic smooth muscle cells (raSMC) and porcine aortic endothelial cells (PAEC)	↑ (PAEC) ↓ (raSMC)	Reveal central role for the endothelin system in stretch-induced apoptosis of the smooth muscle cells. ET-1 binding to the ET_B_ receptor subtype results in apoptosis rather than proliferation	Cattaruzza et al., 2000, 2001.
Activin	Keratinocytes, Fibroblasts, Inflammatory cells, Macrophages	Normal and wounded skin	↑	Stimulates keratinocyte migration, fibroplasia, and matrix production	Hübner et al., 1996

*↑, increased in response to mechanical strain; ↓, decreased in response to mechanical strain; ↑↑, overexpression in response to mechanical strain; II, unchanged in response to mechanical strain*.

Among the growth factors families, the EGF family and the TGF-β family are thought to play central roles (Hashimoto, 2000) and they provide dual-mode regulation of keratinocyte growth via the proliferation-stimulating effect of EGF and the proliferation-inhibiting effect of TGF-β (Amendt et al., 2002; Secker et al., 2008). Although, these growth factors appear to share several downstream pathways of cell membrane molecules, the direct effects of mechanical stress on TGF and EGF are yet to be investigated (Takei et al., 1998). Although, human epidermal keratinocytes express ErbB1, ErbB2, and ErbB3, they do not express ErbB4 (Hashimoto, 2000). Similarly, signals originating from ErbB1 play crucial roles in mediating the pro-survival and proliferative programs of keratinocytes (Shirakata et al., 2010). The expression of cadherins, integrins, and various other ECM components that contribute to the maturation of new blood vessels are regulated by FGF2 (Cross and Claesson-Welsh, 2001). HB-EGF shows a starring role in the reepithelialisation and granulation tissue formation (Marikovsky et al., 1996). The strongest autocrine stimulation to cell growth is provided by amphiregulin (Piepkorn et al., 1994).

## Ion channel related to mechanical strain

Mechanical stress to the cell surface activates the mechanosensitive ion channels along with other membrane-associated signal-transduction molecules (De Filippo and Atala, 2002; Wang et al., 2009). The precise mechanism of activation and modulation of ion channels by mechanical forces that results in biologically meaningful signals are subjects of intensive research (Martinac, 2014). Sachs (1991) reported that, in order to make conformational changes of a channel, external forces must do work on the channel and be dominated by the distance the force move. Howard and Hudspeth (1988) estimated that the stress activated channels change their dimensions by 4 nm between the closed and open states. These stretch-induced ion channels are mainly cation (Ca^2+^, K^+^, and Na^+^) channels and a few anion (Cl^−^) channels (Jackson, 2000; Nilius and Droogmans, 2001).

The vast majority of channels open because of the changes in lipid bilayer, membrane fluidity or tension and are regulated by voltage, extracellular ligands, phosphorylation, influx of Ca^2+^ and direct (physical interactions between G-protein subunits and the channel protein) or indirect (via second messengers and protein kinases) interaction with activated G proteins (Christensen, 1987; Maroto et al., 2005; Lumpkin and Caterina, 2007; Hahn and Schwartz, 2009). The mechanosensitive activities of ion channels are cell dependent and vary from cell to cell (Hsieh and Nguyen, 2005). The elevated intracellular Ca^2+^ levels are cytotoxic and provide the apoptotic stimulus in multiple cell types. The studies of past decades indicated the involvement of different ions in stretch induced response and cytoskeleton are also associated (Jackson, 2000; Wang et al., 2001). However, the precise ion channels related mechanisms for tissue expansion are yet to be studied.

## Second messengers system in strain-induced responses

The exact role of second messengers system in response to tissue expansion (i.e., epithelial cell proliferation) is not clearly elucidated (De Filippo and Atala, 2002). Several investigations in last decades of the past century reported that, cyclic adenosine monophosphate (cAMP) plays an important role to influence cell growth, differentiation, proliferation and protein synthesis depending on the source of cells and experimental conditions (Bang et al., 1992; Florin-Christensen et al., 1993; Zhang et al., 2016). Takei et al. (1997) found significant increase of protein production in keratinocytes subjected to cyclic strain. Moreover, net collagen amount decreases when the levels of cAMP in skin fibroblasts is increased. Study of Acute and chronic cyclic strain reduces adenylate cyclase activity in cultured coronary vascular smooth muscle cells that could promote strain-induced cell contraction (Wiersbitzky et al., 1994). The findings of previous researches on second messengers are listed in the Table 3.

**Table 3:** **Effects of mechanical strain on major second messengers**.

**Second messenger**	**Experimental condition (expansion or stress)**	**Effects on second messenger**	**Major observation**	**References**
Cyclic adenosine monophosphate (cAMP)	Cyclical elongation and relaxation of smooth muscle cells grown on elastic membrane	↑	Collagen production inhibited by raised cAMP.	Kollros et al., 1987
	Round tissue expanders were placed dorsally	↓	Protein production increased in expanded tissue	Johnson et al., 1988
	Constant and cyclic strain (150 mmHg for 5 days) of human keratinocytes	↓	Protein production significantly increased	Takei et al., 1997
Prostaglandin E2 (PGE2)	Cyclical elongation and relaxation of smooth muscle cells grown on elastic membrane	↑	Collagen production inhibited by increased PGE2.	Kollros et al., 1987
	Constant and cyclic strain (150 mmHg for 5 days) of human keratinocytes	↓	Protein production significantly increased	Takei et al., 1997
Phosphodiesterase IV (PDE IV)	Constant and cyclic strain (150 mmHg for 5 days) of human keratinocytes	↑	Controll cAMP levels in human keratinocytes	Takei et al., 1997

*↑, increased in response to mechanical strain; ↓, decreased in response to mechanical strain*.

Inositol phosphate (IP), c-fos, and phospholipids (PL) are thought to mediate extracellular signals to the nucleus but the precise mechanisms need further reaserch (Takei et al., 1998). Moreover, Molinari (2015), proposed hydrogen ion (H^+^) as a second messenger to mediate Ca^2+^ mobilization especially in IP3/Ca^2+^ signaling pathway. At the beginning of 21st century, Buscà et al. (2000) reported that the BRAF gene (which mediates growth signaling at a level just below RAS) can be activated by cAMP in melanocytes. Extracellular signals (growth factors) that activate G-protein-couples receptor can result in the activation of adenylate cyclase to upregulate cAMP leading to the activation of RAS and further activation of BRAF and the downstream cascades (Simonds, 1999; Davies et al., 2002; Pollock and Meltzer, 2002). Likewise the studies on second messengers have been done on different cell lines, this study was also performed with cultured cell lines derived from human tumors, so further investigations are needed to be executed with expanded tissue and acutely stretched skins to determine the precise roles of the ubiquitous and archetypal intracellular second messengers.

## Conclusion and future perspectives

In this article, recent advances in tissue expansion in the field of plastic and reconstructive surgery were described with a special focus on the biological response and the activated pathways leading to either proliferation or apoptosis. Emphasis was given on the roles of membrane bound molecules such as integrins, G-protein, growth factors, stretch-activated ion channels, and secondary messengers. Although, studies of past decades demonstrated that, mechanical stimulation is capable to activate highly integrated signaling cascades resulting in the new skin production, questions remain on how different types of stimulation works on, different cells following different signal transduction pathways. For example, studies on the cells from the kidney differ significantly compared to the cells of skin which are subjected to constant expansion or mechanical forces. Moreover, studies using cultured cells rather than intact tissue (skin) cannot clarify the exact effects of tissue expansion. Similarly, stimulus such as shearing, heat, and shock cannot provide natural microenvironment to better understand how cell adapt to changes during tissue expansion. Furthermore, the signaling pathways activated by different biochemical factors were investigated in linear methods such as single pathway analysis, which is insufficient to describe multiple signaling pathways involved in cell proliferation and/or apoptosis. Therefore, in depth comparative proteomic and genomic analysis with expanded tissue or acutely stretched skin would reveal the pathways and molecules responsible for cell proliferation and/or apoptosis ultimately skin regeneration.

## Author contributions

Concept development: MTR. Writing the manuscript: MAR, MTR, MSH, ZR, NY, and JC. Literature review for data collection: MAR, MTR, MSH, and ZR. Figure and Tables: MAR, MTR, MSH.

### Conflict of interest statement

The authors declare that the research was conducted in the absence of any commercial or financial relationships that could be construed as a potential conflict of interest.
